# Automated Patch Clamp for the Detection of Tetrodotoxin in Pufferfish Samples

**DOI:** 10.3390/md22040176

**Published:** 2024-04-15

**Authors:** Mònica Campàs, Jaume Reverté, Àngels Tudó, Mounira Alkassar, Jorge Diogène, Francesc X. Sureda

**Affiliations:** 1 IRTA, Marine and Continental Waters (AMiC) Programme, Ctra. Poble Nou del Delta, km. 5.5, 43540 La Ràpita, Spain; jaume.reverte@irta.cat (J.R.); mounira.alkassar@irta.cat (M.A.); jorge.diogene@irta.cat (J.D.); 2 Department of Basic Medical Sciences, Universitat Rovira i Virgili, C/Sant Llorenç 21, 43201 Reus, Spain; angels.tudo@urv.cat

**Keywords:** tetrodotoxin, Neuro-2a cells, automated patch clamp, pufferfish

## Abstract

Tetrodotoxin (TTX) is a marine toxin responsible for many intoxications around the world. Its presence in some pufferfish species and, as recently reported, in shellfish, poses a serious health concern. Although TTX is not routinely monitored, there is a need for fast, sensitive, reliable, and simple methods for its detection and quantification. In this work, we describe the use of an automated patch clamp (APC) system with Neuro-2a cells for the determination of TTX contents in pufferfish samples. The cells showed an IC_50_ of 6.4 nM for TTX and were not affected by the presence of muscle, skin, liver, and gonad tissues of a *Sphoeroides pachygaster* specimen (TTX-free) when analysed at 10 mg/mL. The LOD achieved with this technique was 0.05 mg TTX equiv./kg, which is far below the Japanese regulatory limit of 2 mg TTX equiv./kg. The APC system was applied to the analysis of extracts of a *Lagocephalus sceleratus* specimen, showing TTX contents that followed the trend of gonads > liver > skin > muscle. The APC system, providing an in vitro toxicological approach, offers the advantages of being sensitive, rapid, and reliable for the detection of TTX-like compounds in seafood.

## 1. Introduction

Marine toxins are a broad group of compounds that may be present in fish, shellfish, and other marine organisms and accumulate throughout food webs, putting consumers at risk. These toxins are byproducts of the metabolism of several microorganisms like microalgae and bacteria and have different mechanisms of action. One of the most toxic marine toxins is tetrodotoxin (TTX), which is thought to be produced by several bacteria species [1,2]. Tetrodotoxin is a water-soluble molecule with a low molecular weight (319.27 g/mol), consisting of guanidinium moiety connected to a highly oxygenated carbon backbone with a 2,4-dioxaadamantane portion containing five hydroxyl groups [3]. Up to now, more than 30 TTX analogues have been described. Tetrodotoxins are usually found in pufferfish (from the family Tetraodontidae), although their presence has also been reported in other marine species and terrestrial animals, such as gastropods, newts, crabs, frogs, sea slugs, starfishes, blue-ringed octopuses, and ribbon worms [4,5,6]. This toxin is responsible for thousands of intoxications every year in Japan and China, where fugu, a delicacy fish only served in selected restaurants, is consumed. However, intoxication cases have also been described in other countries [7]. For instance, the 37th Annual Report of the American Association of Poison Control Centers’ reported 210 cases of TTX poisoning in the USA [8]. In Europe, only one case has been registered after the ingestion of shellfish (*Charonia lampas lampas*) [9,10] in Spain. Nevertheless, in the Mediterranean Sea, where 13 species of pufferfish have been reported [11], the presence of toxic pufferfish is increasing in part due to the invasion of *Lagocephalus sceleratus* through the Suez Canal [12], which was first reported in Turkey in 2003 [13,14].

To avoid intoxications, the Japanese government established a regulatory limit of 2 mg TTX equiv./kg of tissue in pufferfish [15]. In the European Union, fishery products derived from the family Tetraodontidae must not be placed on the market (Regulation (EC) No. 854/2004) [16]. Regarding its presence in shellfish, the CONTAM panel of the European Food Safety Authority (EFSA) concluded that a concentration lower than 44 µg TTX/kg of shellfish meat and/or the equivalent toxic amount of its analogues, based on a portion size of 400 g, is not expected to result in adverse effects in humans [17].

Like for other marine toxins, the mouse bioassay (MBA) was initially implemented for the detection of TTX in food. Due to its low specificity and ethical concerns, other methods have been developed. Instrumental analysis techniques like high-performance liquid chromatography coupled to fluorescence detection (HPLC-FLD) and liquid chromatography coupled to mass spectrometry (LC-MS/MS) attain low limits of detection (LODs) [18]. However, they are subject to limitations like complexity and costs both derived from the required expensive equipment and trained personnel. Availability of standards can also be a critical issue. In these instrumental analysis techniques, the detection is based on the chemical structure of the compounds. On the contrary, cell-based assays (CBAs) are based on the effect of the toxin and its mechanism of action and, therefore, provide an estimation of the toxicity of a sample. Typically, neuroblastoma cell lines have been used to detect toxins of marine origin [19,20]. This is due to the presence in the cells of several targets that are normally affected by marine toxins and that may cause a loss in function or cell death, the endpoint of which is usually measured in many methods [21,22,23]. Among them, Neuro-2a cells are a particular type of neuroblastoma from mice that has been used for TTX detection [24,25]. Tetrodotoxins, acting through the blockade of voltage-gated sodium channels (VGSCs, also known as Na_v_ channels) that are present in Neuro-2a cells, prevent the lethal effect of ouabain and veratridine, two drugs that evoke the continuous depolarisation of these cells through different mechanisms and ultimately their death, which is detected using a colorimetric method [19]. Unfortunately, the CBA also requires trained personnel and is time-consuming, as several days are needed to detect toxicity from a sample. Other analysis methods include immunoassays and aptamer-based assays, as well as immunosensors and aptasensors, based on antibodies and aptamers, respectively, and therefore recognising chemical structure and not toxicity [26,27,28,29,30].

Effect on ion channels is a common feature of some marine toxins [31] and, as mentioned before, TTX and analogues act through the blockade of Na_v_ channels. This mechanism of action has been thoroughly studied using patch clamp electrophysiological assays, where the membrane potential of a cell or a portion of a nerve is controlled, and the ionic currents are recorded. As patch clamp experiments are delicate and require specifically trained personnel, several attempts have been pursued to make electrophysiological experiments more accessible to general laboratory personnel and, most importantly, more efficient in terms of high-throughput analysis. This has been achieved by replacing the patch clamp pipette with a planar glass chip wherein the cell is immobilised, avoiding the use of microscopes and micromanipulators, and providing a complete robotised system, called automated patch clamp (APC). This system has been widely used since the 2000s by the pharmaceutical industry in screening studies for pharmacological activity and in preclinical toxicology. Regarding its application to marine toxins, several groups have explored the use of APC to study the mechanism of action of azaspiracids (AZAs) [32], ciguatoxins (CTXs) [33], and brevetoxins (PbTxs) [34], and to determine toxicity equivalency factors (TEFs) of paralytic shellfish poisoning (PSP) toxins [35]. Although TTX has already been detected in an APC system [36], Neuro-2a cells, which are the gold standard in CBAs, have never been used in APC systems. Furthermore, fish samples containing TTX have never been analysed in APC systems either. In this work, Neuro-2a cells have been used in an APC system and tested for TTX detection. The possible pufferfish matrix effects both on the Na_v_ function and on the blockade by TTX have been evaluated using tissue extracts from a *Sphoeroides pachygaster* specimen (TTX-free). The APC system was then applied to the determination of TTX contents in tissue extracts from a *L. sceleratus* specimen (TTX-containing specimen) and the results were compared to those obtained with CBAs, as well as instrumental analysis and immunochemical techniques from a previous work. This is the first attempt to evaluate the applicability of APC with Neuro-2a cells and to detect TTX in naturally contaminated fish samples.

## 2. Results

### 2.1. Suitability of Neuro-2a Cells for TTX Detection Using APC

**Figure 1:**
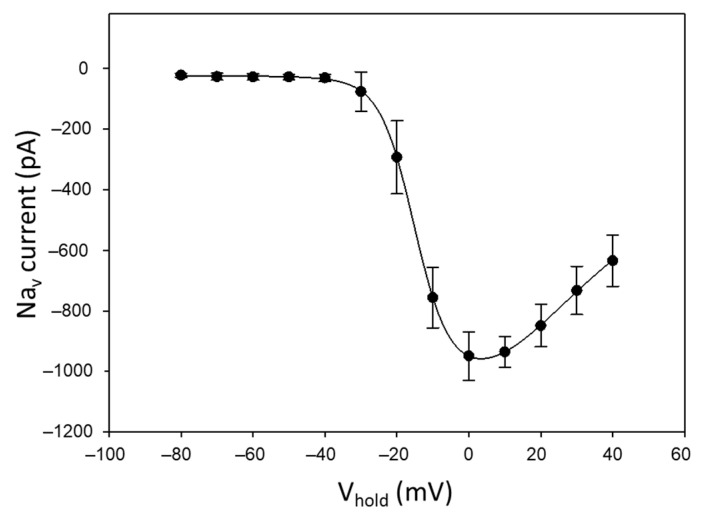
Intensity of Na_v_ currents in Neuro-2a cells at different applied membrane potentials prior to the exposure to VGSC blockers (*n* = 4).

With the aim of verifying the suitability of our Neuro-2a cells for the detection of TTX with APC, the electrophysiology activity of these cells was initially assessed prior to toxin exposure. Once the whole-cell configuration was achieved (with sealings higher than 1 GΩ), the Neuro-2a cells used in this work showed a half-activation potential (V_half_) of −16 mV and a peak current of −950 pA (Figure 1).

**Figure 2:**
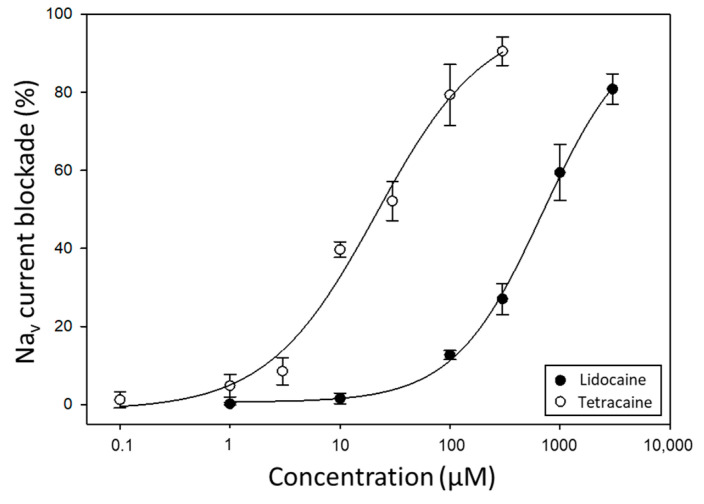
Dose–response curves for lidocaine and tetracaine showing the blocking effect of these compounds on Na_v_ currents in Neuro-2a cells (*n* = 4).

Then, the response of these cells to compounds with VGSC-blocking activity was assessed. First, the blocking activity of lidocaine and tetracaine was evaluated. A dose-dependent reduction in Na_v_ currents was observed by the effect of these two well-known anaesthetics used as drugs for relieving local pain (Figure 2). Lidocaine inhibited Na_v_ currents with an IC_50_ of 717.2 µM, whereas tetracaine showed a higher blocking activity with an IC_50_ of 22.5 µM.

**Figure 3:**
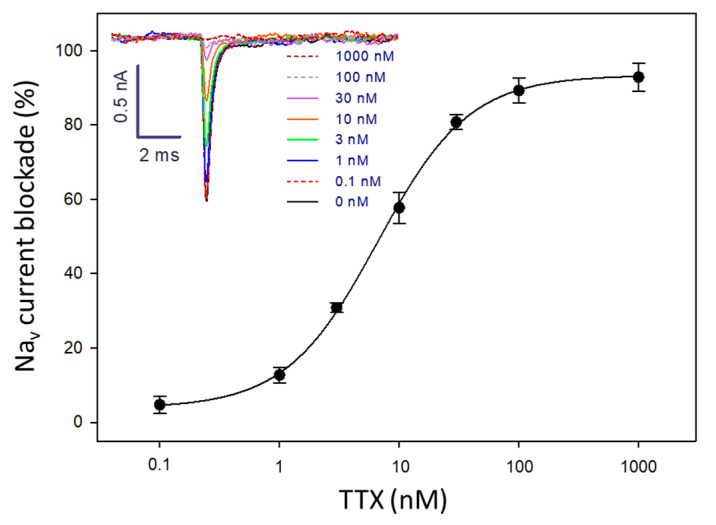
Dose–response curve for TTX showing the blocking effect of this toxin on Na_v_ currents in Neuro-2a cells (*n* = 4). Inset: traces from APC recordings at different TTX concentrations.

Next, the blocking activity of TTX was evaluated. The Na_v_ currents in Neuro-2a cells were completely inhibited when perfusing the system with a solution of TTX at 100 nM. This blockage could be reversed by injecting external solution into the APC chip (system regeneration), indicating that the effect of TTX on the electrophysiological activity of the VGSCs is transient. After regeneration, the response of the Neuro-2a cells to TTX was still the same, allowing for the reuse of the system multiple times as long as the sealing and the membrane integrity remained stable (no leakage currents). From the dose–response curve for TTX, an IC_50_ value of 6.4 nM was obtained (Figure 3).

The fact that Neuro-2a cells have shown an electrophysiological activity close to other cell types also used in the patch clamp together with the good sensitivity to VGSC blockers suggested their suitability as a cellular model for the analysis of samples potentially containing TTX with APC.

### 2.2. Evaluation of Pufferfish Matrix Effects on Na_v_ Function and Blockade by TTX

**Figure 4:**
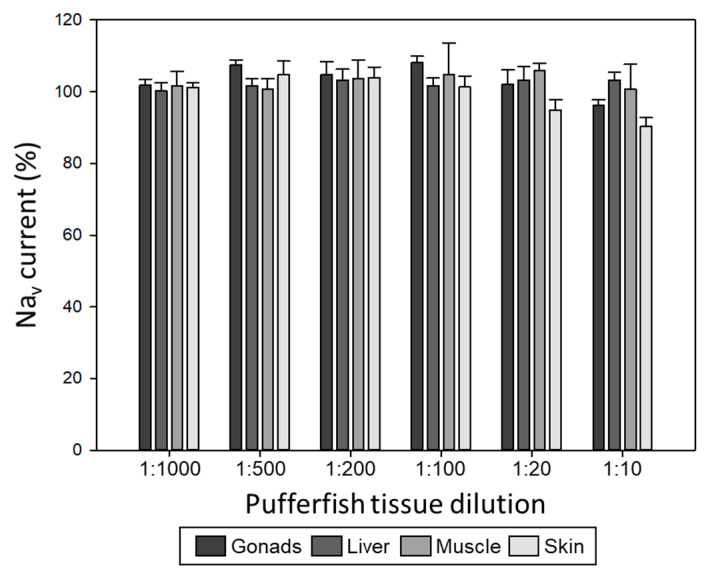
Effect of different dilutions of different pufferfish tissue extracts from a *S. pachygaster* specimen (TTX-free) on Na_v_ currents in Neuro-2a cells (n = 4).

The analysis of compounds of interest in biological samples can be affected by the presence of components other than the analyte. To study the matrix effects in Na_v_ currents from Neuro-2a cells, we evaluated the effect of different dilutions of tissue extracts (muscle, skin, liver, and gonads) from a *S. pachygaster* pufferfish specimen on the system with immobilised Neuro-2a cells. The extracts were previously tested for the presence of TTX by LC-MS/MS and were considered negative [37], as well as by CBAs. Experiments showed that the Na_v_ currents start to decrease at the highest matrix concentration (1:10 dilution) for skin and gonads, whereas for muscle and liver, the VGSC activity remained stable (Figure 4). Nevertheless, this inhibition of the signal was never higher than 20%, since VGSC activity was always higher than 80%, which is the value used as a threshold in the quantification of TTX contents. For good measure, the 1:20 dilution (10 mg/mL) was chosen as a safe tissue extract dilution to work with in subsequent experiments.

**Figure 5:**
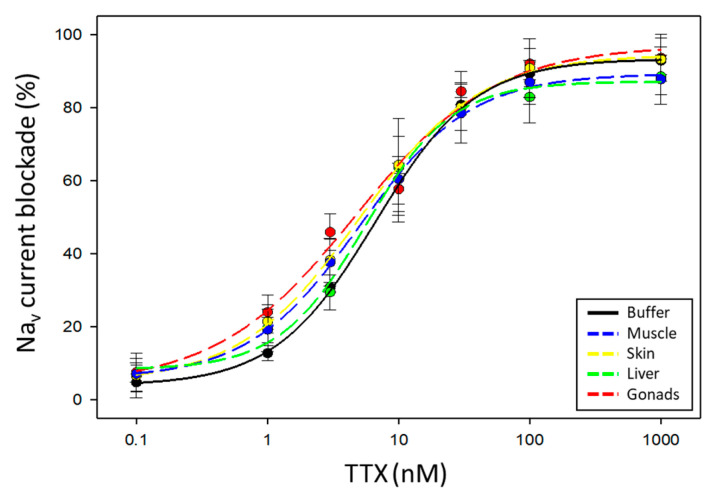
Dose–response curves for TTX in the absence and presence of different pufferfish tissue extracts from a *S. pachygaster* specimen (TTX-free) at 1:20 dilution (n = 4).

Even though the tissue is used at a concentration that does not interfere with the Na_v_ current recordings in Neuro-2a cells, we cannot exclude that matrix compounds do not interfere on the blockade action of VGSCs by TTX. Therefore, to evaluate this potential interference, pufferfish tissue extracts (at 1:20 dilution) of the *S. pachygaster* specimen (TTX-free) were spiked with serial concentrations of TTX. Each dose–response curve was fitted to a sigmoidal four-parameter logistic curve (Figure 5), and analytical parameters were obtained. In Table 1, the Hill slope (slope of the sigmoidal curve between the two plateaus), IC_50_ (half-maximal inhibitory concentration), and *R*^2^ (coefficient of determination) values of the five equations are detailed. No significant differences among Hill slopes or among IC_50_ values from the dose–response curves for the five groups (buffer and four tissues) were obtained (*p* value > 0.05). Therefore, the data revealed that, at this matrix concentration, the compounds from the tissues of *S. pachygaster* used as a TTX-free specimen do not interfere with the TTX calibration curve and proved that the assay could be applied to the analysis of TTX in pufferfish samples. Additionally, recovery being 100%, there is no need to apply any recovery factor in the determination of TTX contents.

The cells used in the APC system showed a working range of 0.05–1.00 mg TTX equiv./kg (between IC_20_ and IC_80_). The repeatability and intermediate precision at 0.15, 0.30, and 0.60 mg TTX equiv./kg were evaluated by performing multiple measurements on the same day and different days, respectively. The relative standard deviation (RSD) values for the measurements performed on the same day were 14, 11 and 10% (N = 4) for 0.15, 0.30, and 0.60 mg TTX equiv./kg, respectively. The RSD values for the measurements performed on different days were 12, 9, and 5% (N = 4) for 0.15, 0.30, and 0.60 mg TTX equiv./kg, respectively. According to AOAC, the system showed appropriate reliability, as the Horwitz ratio (HorRat) values (ratio of the RSD calculated from the data to the RSD predicted from the Horwitz equation, which is 11% when the analyte concentration is 1 ppm) were between 0.5 and 1.3 (and are considered acceptable between 0.5 and 2) [38,39].

### 2.3. Analysis of TTX in Lagocephalus Sceleratus Samples

The APC system with Neuro-2a cells was then used to quantify the TTX contents in different tissues of a *L. sceleratus* pufferfish specimen (Table 2). Additionally, extracts were analysed by a CBA recently developed by our group [25]. The extracts had also been analysed by several techniques in previous works: LC-MS/MS, liquid chromatography coupled to high-resolution mass spectrometry (LC-HRMS), maleimide-based enzyme-linked immunosorbent assay (mELISA) [37], electrochemical immunosensor, and optical surface plasmon resonance (SPR) immunosensor [40].

The APC system revealed the presence of TTX equiv. in all extracts, with contents above the Japanese regulatory limit of 2 mg TTX equiv./kg for all tissues except for muscle. Gonads was the most toxic tissue, followed by liver, skin, and muscle. The TTX levels in these tissues were also determined using the CBA and the results followed the same trend as that provided by the APC: gonads > liver > skin > muscle.

## 3. Discussion

The use of primary cell cultures freshly initiated from animal tissues for electrophysiology studies using patch clamp has been widely explored in the literature [41,42]. Due to their origin, the distribution of VGSCs on the membranes of these cells is highly conserved, and this is interesting from an analytical point of view since they may provide toxicological responses closely related to the ones suffered in real poisoning events. However, the difficulties associated with cell culturing added to the dependence on animals as a source of cells from primary cultures reduce their applicability [23], especially when high throughput analysis is pursued and high cellular densities are required. Contrarily to primary cell cultures, immortalised cell lines may not express all receptors present in vivo. Nonetheless, cancer cell lines are certainly excellent models when the target receptors fit the purpose of a specific research [43]. In recent decades, there has been a tendency towards the use of immortalised cell lines for patch clamp due to their simplicity in terms of handling and their high growth rate. At the physiological level, not all the VGSC subtypes are sensitive to TTX. In fact, from the nine closely related VGSCs known in mammals, only Na_v_1.1, Na_v_1.2, Na_v_1.3, Na_v_1.4, Na_v_1.6, and Na_v_1.7 are sensitive to TTX, whereas Na_v_1.5, Na_v_1.8, and Na_v_1.9 are resistant [44,45]. Additionally, among the TTX-sensitive VGSCs, there are differences regarding the affinity to TTX. Na_v_1.3 is one of the VGSCs with more affinity against TTX [46]. However, not much is known about the affinity of VGSCs against other TTX analogues that may also be present in naturally contaminated samples. For example, Na_v_1.6 has more sensitivity to 4,9-anhydroTTX than Na_v_1.3 [47]. Due to the still missing information regarding all Na_v_ channels and TTX analogues and because the main objective of this work is to demonstrate that APC is a useful bioanalytical tool for the detection of TTX in pufferfish, the use of a cell line with a heterogenic expression of VGSCs for a global electrophysiology assessment was desired. Therefore, we selected Neuro-2a cells as a cellular model for this study.

Neuro-2a cells are an immortalised murine neuroblastoma cell line widely used for toxicological studies due to their ability to endogenously express VGSCs, the target of several marine neurotoxins. The electrophysiological activity of this cell line has been previously studied in other works [48,49,50,51,52]. However, certain discrepancies have been observed with regard to the activation potential required to depolarise the cell and/or the Na_v_ current intensities achieved. This variability in the electrophysiological activity of Neuro-2a cells may be attributed to the heterogenicity of this type of cell cultures, which may display notorious differences in the expression of VGSCs with respect to the parental cell type [53], or even from one cell batch to another. In this work, Neuro-2a cells showed appropriate Na_v_ currents and a V_half_ of activation similar to that reported for human Na_v_1.3 [54]. Even though the peak intensity was lower than in other studies with primary cultures from human hippocampus (around −2000 pA) [55], it was higher than that reached in other works with undifferentiated Neuro-2a cells (−285 pA) [56]. Therefore, according to these results, the Neuro-2a cells showed an appropriate electrophysiological activity on APC without the need of any cell differentiation or sensitisation pretreatment. When lidocaine and tetracaine were evaluated, IC_50_ values were similar to other values already published for cells expressing VGSCs [57,58]. Regarding TTX, complete inhibitions were observed at 100 nM, as also observed with ND7/23 (another murine neuroblastoma cell line) [59], and the IC_50_ value agreed with those reported with other cellular models only expressing Na_v_1.1 [47], Na_v_1.2 [60], Na_v_1.3 [47], Na_v_1.4 [61], Na_v_1.6 [62], or Na_v_1.7 [63]. Compared to lidocaine and tetracaine, TTX had a VGSC-blocking activity more than 10^3^ and 10^5^-fold higher, respectively.

Automated patch clamp cannot distinguish between the different TTX analogues that may be present in a sample, but it provides an excellent approximation of the overall toxicity of that sample due to the presence of VGSC blockers. Nevertheless, as in any analytical technique, matrix effects should be carefully evaluated. Considering that tissue extracts should be diluted at least 20 times (10 mg/mL) and 20% as the minimum value of detectable blockade using this method, which corresponds to 0.5 ng/mL, our system should be able to detect as low as 0.05 mg TTX equiv./kg in pufferfish samples. This value is far below the Japanese regulatory limit of 2 mg TTX equiv./kg. The application of the APC system with Neuro-2a cells to the analysis of different tissues of a *L. sceleratus* pufferfish specimen provided the TTX equiv. contents in those samples. The TTX levels quantified in *L. sceleratus* tissues were in the range of those obtained in other studies: 0.05−58.44, 0.1−35.05, 0.09−1380.8, and 0.17−8248.51 mg TTX equiv./kg in muscle, skin, liver, and gonads, respectively [25]. The high TTX contents in the liver and/or gonads of pufferfish have already been observed in other works [12,25,64,65,66,67,68]. Regarding the high TTX contents found in the liver, Anastasiou and co-workers suggested that it is related to hepatic uptake [66]. As also reported in other studies [69,70,71], TTX is initially accumulated at high concentrations in the liver, then transferred to the skin of males or gonads of females. The *L. sceleratus* specimen analysed in this work was a female. Many studies on different pufferfish species attribute the high TTX concentrations in gonads to the enhancement of the reproduction success and the role of TTX as a male-attracting pheromone [64,65,68,72].

The CBA previously developed by our group is also based on the effect of TTX on Neuro-2a cells and it also provides a composite toxicological response from a sample. However, the variable used to measure the toxicological effect is different. Whereas in the APC the Na_v_ currents are recorded, in this CBA cell viability is monitored using the MTT dye. As previously mentioned, in the CBA TTX counteracts the toxic effect of ouabain and veratridine (which induce cytotoxicity), resulting in higher cell viability with respect to the controls without TTX. The fact that APC and CBA measure different variables to estimate toxicity may explain that the determined TTX contents are slightly different between these two methods for some tissues; for example, the liver. It is important to note that the liver was the tissue with the highest fat content and that required an additional liquid-liquid partition with hexane for the purification of the extract. Therefore, the effect of liver matrix compounds on the response cannot be completely ruled out. In fact, this tissue is the one that shows the greatest variability among analytical techniques. While the APC and the CBA provide a toxicological response, each one has its own advantages and limitations. In the APC, the VGSC blocking activity is evaluated with non-treated cells. On the contrary, the CBA requires the use of ouabain and veratridine, which represent an additional stress in the cells previous to the exposure to the extracts and more controls. The CBA requires 48 h (24 h for cell immobilisation on plates and 24 h for toxin exposure and cell viability measurement), whereas in the APC, results can be obtained in less than 30 min. On the other hand, the CBA is usually performed on 96-well plates, whereas the chips used in this work contain only eight chambers, which nevertheless is more than in conventional patch clamp. Still, APC systems have already been designed to record up to 384 cells in parallel. Additionally, in the APC system, the TTX blocking activity can be reversed by perfusing the cells with external solution, restoring the sodium currents, and therefore making it possible to regenerate and reuse the system.

When comparing the TTX contents obtained from these two toxicological approaches with the results provided by LC-MS/MS and LC-HRMS, it is important to take into account that these instrumental analysis techniques determine individual TTX and TTX analogue contents based on their chemical structures, not their toxicities. Therefore, the use of TEFs plays a crucial role in translating the TTX analogue data into global toxicity estimations, expressed as TTX equivalents (Table 1). Ideally, when comparing both techniques, TEFs derived from APC should be applied to TTX analogues. However, the relative toxicity of TTX analogues has not been determined by APC yet. In this work, TEFs previously obtained by the CBA [25] have been applied: 0.75, 0.404, 0.139, and 0.011 for 5,11-dideoxyTTX, 11-norTTX-6(*S*)-ol, 11-deoxyTTX, and 5,6,11-trideoxyTTX, respectively. Additionally, TEFs for 4-*epi*TTX, 11-norTTX-6(*R*)-ol, 4,9-anhydro-5,6,11-trideoxyTTX, and 4,4a-anhydro-5,6,11-trideoxyTTX were assumed to be those of their corresponding isomeric analogues. For 4,9-anhydroTTX and 5-deoxyTTX, not investigated in that work, a TEF = 0 was assumed, since trials with MBA resulted in very low toxicities [73,74]. The results obtained by instrumental techniques, after applying the corresponding TEFs, followed the same trend as those provided by APC, gonads being the most toxic tissue followed by liver, skin, and muscle. However, as also observed when comparing with CBAs, a considerable difference was observed in the TTX contents of the liver extract between APC and instrumental analysis methods. The presence of unknown TTX analogues that have not been quantified or liver matrix compounds could be responsible for this discrepancy. On the other hand, it is important to state that both the CBA and the APC provide an estimation of TTX contents resulting from their initial interaction with the VGSCs. Hence, the potential presence in the studied samples of other compounds acting directly on these receptors such as other VGSC blockers or VGSC activators may also interfere with the TTX estimations.

Immunochemical techniques provide a global response from all TTX and TTX analogues that cross-react with the antibody. Again, each TTX analogue cross-reacts with a higher or lower structural affinity, contributing at a higher or lower extent to the global response [23,75]. The TTX contents obtained with the APC were of the same order of magnitude as those provided by the immunochemical approaches. Slight differences between immunosensing and toxicity approaches may be attributed to differences between cross-reactivity and toxicity factors of the present TTX analogues and/or the matrix effects that may interfere in the quantifications obtained with the immunosensing tools.

## 4. Materials and Methods

### 4.1. Reagents and Equipment

Tetracaine hydrochloride and lidocaine hydrochloride monohydrate were obtained from Merck KGaA (Darmstadt, Germany). Tetrodotoxin standard was purchased from Tocris Bioscience (Bristol, UK) and the standard solution was prepared at 1 mg/mL in 1% acetic acid. The automated patch clamp Patchliner with 8 amplifier channels, 2 HEKA EPC10 Quadro amplifiers, NPC-16 borosilicate recording chips (medium resistance), external solution (140 mM NaCl, 4 mM KCl, 1 mM MgCl_2_, 2 mM CaCl_2_, 5 mM D-glucose monohydrate, 10 mM HEPES/NaOH, pH 7.4), internal solution (50 mM CsCl, 10 mM NaCl, 60 mM CsF, 20 mM EGTA, 10 mM HEPES/CsOH, pH 7.2), and seal enhancer solution (10 mM HEPES, 130 mM NaCl, 5 mM glucose, 4 mM KCl, 10 mM CaCl_2_, 1 mM MgCl_2_, pH 7.4, MOsm 302) were obtained from Nanion Technologies GmbH (Munich, Germany). Neuroblastoma murine (Neuro-2a) cells were purchased from ATCC LGC standards (Manassas, VA, USA). Foetal bovine serum (FBS), penicillin/streptomycin solution, phosphate-buffered saline (PBS), Roswell Park Memorial Institute (RPMI) medium, sodium pyruvate, trypsin-EDTA enzyme, ouabain, veratridine, and thiazolyl blue tetrazolium bromide (MTT) were purchased from Merck KGaA (Darmstadt, Germany).

### 4.2. Pufferfish Samples and Tetrodotoxin (TTX) Extraction

Fish extracts were obtained from a previous work [54]: one blunthead pufferfish (*Sphoeroides pachygaster*, Müller and Troschel, 1848) (TTX-free specimen) and one silver-cheeked toadfish (*Lagocephalus sceleratus*, Gmelin, 1789) (TTX-containing specimen) caught in 2014 in Dénia (Alicante, Spain).

The two pufferfish specimens were dissected, and the muscle, skin, liver, and gonads were retrieved. A double TTX extraction was performed as previously described [76]. Initially, 10 g of pufferfish tissue was weighed in 50 mL tubes and mixed with 25 mL of 0.1% acetic acid. After vortexing for 2 min at 2500 rpm, the tubes were immersed in a water bath set at 100 °C for 10 min with occasional stirring. Then, the tubes were cooled down and centrifuged at 2500 rpm for 5 min at 4 °C. The supernatants were collected, and a second extraction was performed with 20 mL of 0.1% acetic acid. The supernatants from the two extractions were pooled and, for the liver samples, an additional liquid–liquid partition with hexane (1:1) was performed to remove fats. The final volume was set to 50 mL, obtaining extracts at a matrix concentration of 200 mg equiv. of pufferfish tissue/mL. All extracts were kept at −20 °C until required. The pufferfish tissue extracts were analysed by liquid chromatography-tandem mass spectrometry (LC-MS/MS), with an LOD and a limit of quantification (LOQ) of 0.05 mg/kg and 0.1 mg/kg, respectively (Rambla-Alegre et al. 2017 [37]).

### 4.3. Neuro-2a Cell Line Maintenance

Cells were maintained in RPMI-1640 medium with L-glutamine solution, supplemented with 10% heat-inactivated FBS, penicillin/streptomycin solution at 0.01 mg/mL and 10 U/mL, respectively, and 1 mM sodium pyruvate in an incubator (BINDER GmbH, Tuttlingen, Germany) at 37 °C in 5% CO_2_ humid atmosphere. The cells used in this work were between the passages 245 and 255.

### 4.4. Automated Patch Clamp Recording

The electrophysiological changes on Neuro-2a cells were recorded using the Patchliner (Nanion Technologies GmbH, Munich, Germany), an automated planar patch clamp device that allows for the analysis of up to eight individual cells simultaneously. The analysis with the Patchliner process is as follows: first, the wells from an NPC-16 borosilicate chip (medium resistance) (Nanion Technologies GmbH, Munich, Germany) were filled up with the internal and external solutions. After checking the electrode contacts, Neuro-2a cell suspension at 100,000 cells/mL in RPMI-1640 and external solution (1:1 ratio) were injected into the chip. A single cell was immobilised on the hole located at the bottom of the chip at a holding potential of −30 mV. After the addition of the enhancer solution for cell sealing, the potential was changed to −100 mV. Patching was assumed to be successful when observing a stable sealing along time, and resistance around 1 GΩ.

For the calibration curves, tetracaine, lidocaine, and TTX standard solutions at different concentrations (0.1, 1, 3, 10, 30, 100, and 300 μM for tetracaine; 1, 10, 100, 300 µM, and 1 and 3 mM for lidocaine; and 0.1, 1, 3, 10, 30, 100 nM, and 1 μM for TTX) were injected. For the evaluation of pufferfish matrix effects in the absence of TTX, tissue extracts of a *S. pachygaster* specimen at different dilutions of (1:1000 (0.2 mg/mL), 1:500 (0.4 mg/mL), 1:200 (1 mg/mL), 1:100 (2 mg/mL), 1:20 (10 mg/mL), and 1:10 (20 mg/mL)) were injected. For the evaluation of pufferfish matrix effects in the presence of TTX, tissue extracts of a *S. pachygaster* specimen at 1:20 dilution (10 mg/mL) were spiked with different TTX concentrations (0.1, 1, 3, 10, 30, 100 nM, and 1 μM) and injected. For the determination of TTX contents in pufferfish samples, tissue extracts of a *L. sceleratus* specimen at different dilutions were injected. In all cases, a volume of 15 µL was injected at a flow rate of 30 µL/s. Na_v_ currents were measured by applying 10 mV increments from −80 mV to 40 mV for 20 ms using two EPC Quatro USB amplifier units (8 probes) (HEKA Elektronik, Stuttgart, Germany) controlled and digitalised in real time with the Patchmaster software (Nanion Technologies GmbH, Munich, Germany). Measurements were performed in triplicate.

### 4.5. Colorimetric Cell-Based Assay (CBA)

The colorimetric CBA was performed as in our previous work [25]. Neuro-2a cells were trypsinised and suspended in culture medium (the same as for maintenance but with 5% FBS instead of 10% FBS). Then, Neuro-2a cells were seeded in a 96-well microplate at a density of 35,000 cells/well in 200 µL of culture medium for 24 h at 37 °C in 5% CO_2_ humid atmosphere. Prior to exposure to TTX standard solution or pufferfish tissue extracts, some Neuro-2a cells were pre-treated with 20 µL of an ouabain and veratridine mixture in PBS at final concentrations of 0.125 and 0.2 mM, respectively. Tetrodotoxin standard solution and pufferfish tissue extracts were dried under an N_2_ stream at 40 °C using a TurboVap evaporator (Zymark corp., Hopkinton, MA, USA), reconstituted in culture medium and serially diluted, and 10 µL was added to the wells with and without ouabain/veratridine pre-treatment. After 24 h, cell viability was measured using the MTT assay (Manger et al. 1993). Absorbance at 570 nm was measured with a Synergy LX microplate reader from BioTek (Agilent Technologies, Inc., Santa Clara, CA, USA). Measurements were performed in triplicate.

### 4.6. Statistical Analyses

The dose–response inhibition curve for lidocaine, tetracaine, and TTX were fitted to sigmoidal four-parameter logistic curve models. To evaluate the effect of the tissue (mussel, skin, liver, and gonads) matrix compounds on the blockade caused by TTX, these curves were also fitted to sigmoidal four-parameter logistic curve models, and the Hill slope and IC_50_ values were estimated. Differences among dose–response curves (in the absence and presence of extract of different tissues from *S. pachygaster* at 1:20 dilution) were assessed by comparing the Hill slopes and IC_50_ values of the five groups (buffer and four tissues) using the Kruskal–Wallis test (non-parametric equivalent of the analysis of variance (ANOVA) test), since data were not normally distributed (according to the Shapiro–Wilk test). Differences were considered statistically significant at the *p* value < 0.05. Statistical analyses were performed using the statistical software Prism 5.0 (GraphPad Software Inc, San Diego, CA, USA).

## 5. Conclusions

In this work, Neuro-2a cells were used in a whole-cell configuration APC system, showing appropriate electrophysiological activity. When exposed to TTX, an IC_50_ of 6.4 nM was obtained, similar to those observed with other cells. Regarding the applicability of the APC system to the analysis of pufferfish samples, the presence of muscle, skin, liver, and gonads tissues of a *S. pachygaster* specimen (TTX-free) at 10 mg/mL did not interfere with the assay and an LOD of 0.05 mg TTX equiv./kg was attained, far below the Japanese regulatory limit. The APC system was applied to the analysis of extracts of a *L. sceleratus* specimen, showing TTX contents that followed the trend of gonads > liver > skin > muscle, comparable to that obtained with other techniques. The use of chips with eight chambers and suction for cell immobilisation together with the robotisation of the system makes APC a rapid and simple technique able to perform high-throughput analysis of VGSC blockers such as TTX and to be implemented in food safety monitoring and research programs.

## Figures and Tables

**Table 1:** Hill slope and IC_50_ values obtained from the sigmoidal four-parameter logistic equations for TTX in the absence and presence of different pufferfish tissue extracts from a *S. pachygaster* specimen (TTX-free) at 1:20 dilution (n = 4).

Tissue	Hill Slope	IC_50_ Value (nM)	*R* ^2^
Muscle	0.997 ± 0.068	4.927 ± 2.350	0.9950
Skin	0.910 ± 0.120	5.017 ± 1.587	0.9961
Liver	1.167 ± 0.204	5.118 ± 0.995	0.9886
Gonads	0.801 ± 0.149	4.264 ± 0.668	0.9859
No tissue	1.089 ± 0.097	6.380 ± 0.860	0.9912

**Table 2:** TTX equivalent contents (mg TTX equiv./kg of tissue) in *L. sceleratus* by several techniques, including APC (n = 4) and CBA (n = 3) from this work.

Technique	Muscle	Skin	Liver	Gonads	Reference
APC	1.59	3.32	13.80	22.46	This work
CBA	2.78	3.68	18.53	26.63	This work
LC-MS/MS *	1.43	2.02	3.96	34.62	[37]
LC-HRMS *	1.23	2.42	7.67	39.44	[37]
mELISA	2.53	3.50	28.30	33.55	[37]
Electrochemical immunosensor	1.45	2.11	16.67	33.90	[40]
Optical SPR immunosensor	3.51	4.42	24.82	30.50	[40]

* TTX equiv. contents after the application of TEFs to the different TTX analogues [25].

## Data Availability

Data availability upon request.
